# Primary Kaposiform Hemangioendothelioma of the Humerus: A Case Report

**DOI:** 10.7759/cureus.21262

**Published:** 2022-01-15

**Authors:** Zafar Ali, Syed M Qasim, Farhan Faisal, Gulfam Jameel

**Affiliations:** 1 Histopathology, Shifa International Hospital Islamabad, Islamabad, PAK; 2 Pathology, Shifa Tameer-E-Millat University, Shifa College of Medicine, Islamabad, PAK; 3 Medical School, Shifa Tameer-E-Millat University, Shifa College of Medicine, Islamabad, PAK

**Keywords:** kasabach–merritt syndrome, kaposiform hemangioendothelioma, khe, bone, vincristine

## Abstract

Kaposiform hemangioendothelioma (KHE) is a neoplasm originating mainly from vessels and has a mild proclivity for malignancy. This neoplasm mainly involves somatic soft tissue and retroperitoneum. Histological findings include a nodular arrangement of oval-to-spindle cells containing pale cytoplasm. Vascular spaces are in the form of slit-like channels in which red blood cells are evident. Here, we report the case of a two-year-old male who presented with Erb’s palsy and a mass lesion in the right humerus. Tissue biopsy features were compatible with KHE.

## Introduction

Kaposiform hemangioendothelioma (KHE) is a rare, often deep-seated, vascular neoplasm, mainly found in infants and children. The tumor is characterized by lobular infiltrates of capillaries and spindled endothelial cells. KHE commonly affects the skin, deep soft tissues of extremities, head and neck, trunk, and retroperitoneum; less commonly, the mediastinum, spleen, bone, and testis are affected [1]. KHE may develop life-threatening thrombocytopenia and consumptive coagulopathy, known as the Kasabach-Merritt phenomenon (KMP). The thrombocytopenia occurs because the platelets get entrapped within the neoplastic tissue [2].

Treatment options vary and include surgical excision with wide margins and adjuvant chemotherapy in various combinations. Very few case reports of KHE involving long bones have been published worldwide. Here, we report the first case of primary bone KHE from Pakistan.

## Case presentation

**Figure 1:**
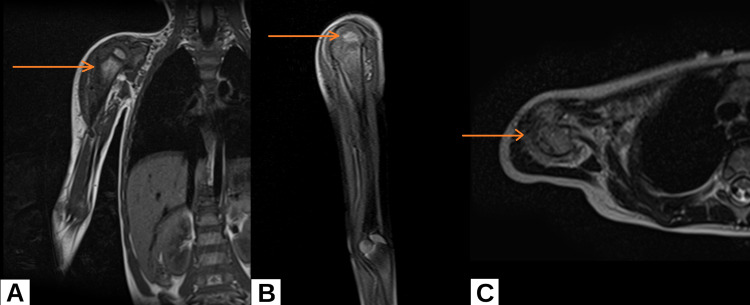
MRI of the patient coronal view (A), sagittal view (B), and transverse view (C). MRI shows an ill-defined, infiltrative, aggressive-looking, hyperintense, enhancing mass arising from the proximal metadiaphysis region of the right humerus with permeative destruction of the cortex and associated periosteal reaction/enhancing soft tissue component. MRI: magnetic resonance imaging

A two-year-old male child presented in the outpatient department with an abnormal position of the right arm. A mass on the shoulder joint was palpated at that time, but no cutaneous changes were seen. The patient appeared to be in no discomfort or pain; however, his parents reported that he pulls the affected arm with his left arm often at night, which may be due to pain. On magnetic resonance imaging (MRI), there was cortical destruction and associated periosteal reaction (Figures 1A-1C). The lesion measured 3.2 × 3.1 × 7.1 cm (anteroposterior × transverse × craniocaudal). A differential diagnosis of Ewing sarcoma and osteomyelitis was made by the radiologist.

**Figure 2:**
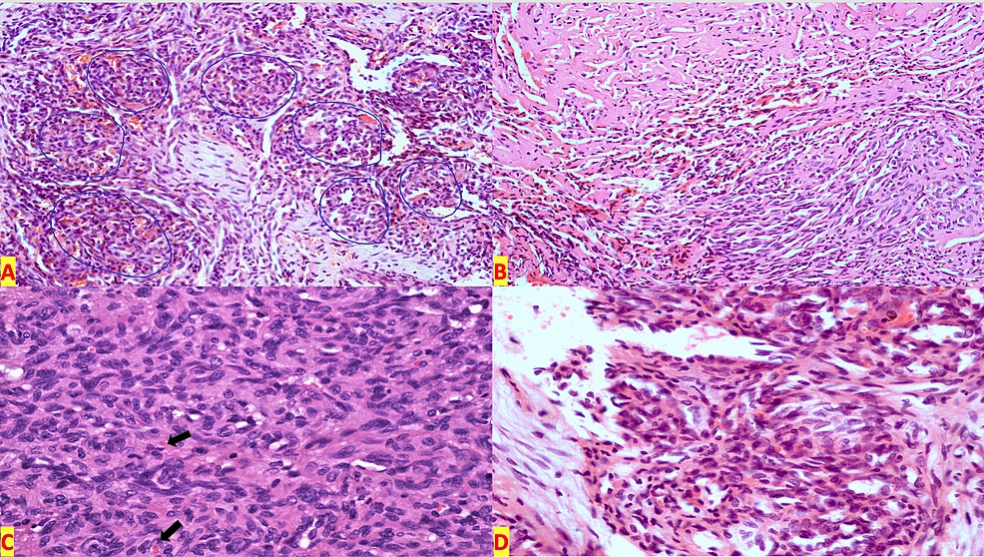
Kaposiform hemangioendothelioma tissue biopsy. A: Low-power view showing glomeruloid aggregates of vascular channels (H/E 10×). B: Slit-like crescentic capillaries within spindle cells (H/E 20×). C: Arrows highlighting fibrin thrombi surrounded by pericytes (H/E 20×). D: RBCs can be seen in the slit-like vessels (H/E 20×). H/E: hematoxylin and eosin; RBC: red blood cell

Tissue biopsy showed bony tissue with a vascular lesion that comprised nodules of spindled endothelial cells forming elongated slit-like, lumina-containing erythrocytes. Curving around these epithelioid nodules were pericytes surrounding platelet-rich microthrombi. No necrosis or mitotic activity was seen (Figures 2A-2D).

**Figure 3:**
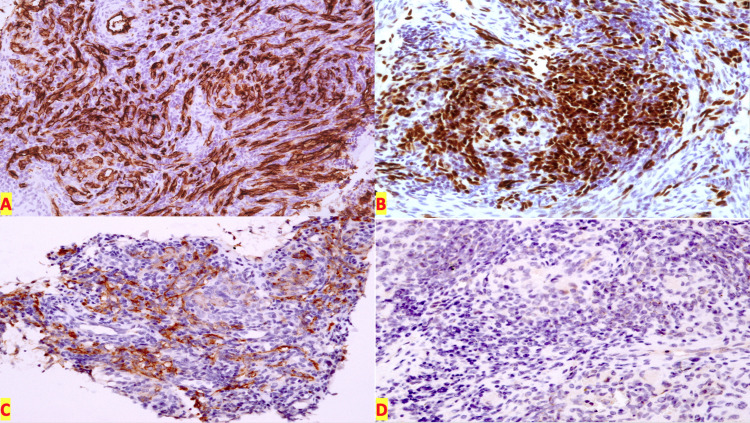
Kaposiform hemangioendothelioma immunohistochemistry. A: CD-34 immunostain highlighting vascular channels (IHC, 20×). B: ERG nuclear staining seen in the tumor cells (IHC, 20×). C: Podoplanin highlighting the vessels and tumor cells (IHC, 20×). D: HHV-8 is negative in the tumor cells (IHC 20×). IHC: immunohistochemistry; ERG: ETS-related gene; HHV-8: human herpesvirus 8

On immunohistochemistry, podoplanin (D2-40) and ETS-related gene (ERG) were positive in neoplastic cells and vascular channels, while CD-99 and human herpesvirus 8 (HHV-8) were negative (Figures 3A-3D).

Based on the morphological features and immunohistochemistry findings, a final diagnosis of KHE was rendered. The patient was referred to the oncologist who started chemotherapy with vincristine.

## Discussion

The term Kaposiform means Kaposi’s sarcoma-like which has spindle pattern cells. KHE is a neoplasm originating mainly from vessels and has a moderate malignant capacity. It can occur in any region such as the retroperitoneum, thoracic spine, appendicular skeleton, and, rarely, visceral organs. In our case, the lesion was present on the proximal end of the right humerus and was involving the bone itself. Although it could be seen on imaging, a definitive diagnosis was made on tissue microscopy and immunohistochemistry. The other differential diagnoses included spindle cell hemangioma, verrucous hemangioma, infantile hemangioma, and Kaposi sarcoma [3]. Ewing sarcoma was the top differential by the radiologist when he saw the scan. A biopsy followed by microscopy and immunohistochemistry was done and a diagnosis of KHE was made based on the findings.

Even when KHE is not associated with any cutaneous lesion, it can be dangerous in terms of clinical outcome and disease progression [4]. KHE is sometimes linked with an entity known as the KMP in which there is entrapment of platelets within the neoplasm, which, in turn, causes thrombocytopenia. According to the literature, KHE with a diameter less than 8 cm is less likely to be associated with KMP [5]. This suggests that the tumors larger than 8 cm have a higher probability of entrapment of platelets and hence causing consumptive coagulopathy. Our patient was not showing any signs and symptoms of KMP which was also confirmed by the coagulation profile of the patient. The other complications of KHE besides KMP include musculoskeletal deformity and compression of vital structures [6].

On MRI, a lesion was seen which was looking aggressively infiltrative and ill-defined and was involving the right humerus. This lesion was seen as a hyperintense mass arising from the metadiaphysis of the right humerus. The cortex of the bone was eroded, and an associated periosteal reaction was present. The lesion measured 3.2 × 3.1 × 7.1 cm (anteroposterior × transverse × craniocaudal). The MRI scan also showed numerous enlarged cervical lymph nodes on the right side. Light microscopy showed irregular nests of undifferentiated vascular channels invading the surrounding tissue. According to the literature, on electron microscopy, incomplete basement membrane and intercellular gaps can be seen [7]. On immunohistochemistry, 96% of cases show immunoreactivity with D2-40. This stain highlights the well-defined glomeruloid cores and neoplastic spindle-shaped cells that are present between the lobules, and the endothelial markers CD31 and CD34 highlight the spindle-shaped cells of vascular channels [8]. Positivity with the above-mentioned immunohistochemical markers was noted in our case. HHV-8 positivity is found in Kaposi sarcoma which is associated with HHV-8 which is tested to rule out the presence of Kaposi sarcoma in cases of suspected KHE [9]. Our patient was HHV-8 negative which ruled out Kaposi sarcoma.

Sirolimus and vincristine are used in the chemotherapeutic management of KHE. The literature shows that sirolimus is very effective in the treatment of KHE [10]. Vincristine was used as a chemotherapeutic agent in our patient. No significant regression was noticed on MRI even though the patient was receiving 0.5 mg vincristine weekly for 12 weeks.

## Conclusions

KHE is a very infrequently occurring neoplasm of vascular origin with moderate malignant potential that may or may not be present along with KMP. This lesion can be present in various areas of the body with or without cutaneous involvement. Hence, it should be kept in mind when differential diagnoses are made in the context of vascular tumors in children.
